# Czech adolescents’ remote school and health experiences during the spring 2020 COVID-19 lockdown

**DOI:** 10.1016/j.pmedr.2021.101386

**Published:** 2021-05-01

**Authors:** Kwok Ng, Alina Cosma, Karel Svacina, Meyran Boniel-Nissim, Petr Badura

**Affiliations:** a School of Educational Sciences and Psychology, University of Eastern Finland, Finland; b Physical Activity for Health Research Cluster, Health Research Institute, Department of Physical Education and Sport Sciences, University of Limerick, Ireland; c Sts Cyril and Methodius Faculty of Theology, Olomouc University Social Health Institute, Palacky, University in Olomouc, Olomouc, Czech Republic; d Faculty of Electrical Engineering and Computer Science, VSB – Technical University of Ostrava, Ostrava, Czech Republic; e Faculty of Physical Culture, Palacký University Olomouc, Czech Republic; f Department of Behavioural Sciences, Kinneret College on the Sea of Galilee, Israel

**Keywords:** Coronavirus, SARS-CoV-2, School closure, Health behaviors, Well-being, Physical activity, Screentime, Food, Sleep

## Abstract

Schools around the world were closed during the spring 2020 lockdown to reduce the spread of COVID-19. As such, these rapid changes to adolescent daily routines may have had immediate as well as long-term effects on their physical, social, and mental health. Therefore, the aim of this study was to examine the experiences, health behaviors and perceived change in health behaviors among adolescents in Czechia during the spring 2020 lockdown. Adolescents (n = 3,440, 54% girls; M_age_ = 13.5 years, SD = 1.6) from all regions of Czechia were recruited to complete a self-report survey based on the Health Behaviour in School-aged Children study, with additional items on household disruptions and socialization during lockdown, as well as items on perceived changes in health behaviors during lockdown. First, we described the self-reported impacts of the spring lockdown by gender and grade. Second, log-linear analyses were used to examine the perceived behavioral changes based on standardized cut-off values reporting for health behaviors and gender. Less than a third, 19% and 32% of the respondents reported economic and psychosocial disruptions to their family life, 79% indicated they had positive opportunities for family interactions or learning new things. Girls and older adolescents reported the worst levels of social and mental well-being. Changes in respondents’ perceived health behaviors varied by reported behavior frequencies and gender. These results may be useful to compare with future secular trends, and may serve as input in developing strategies to counter the impact of the spring 2020 or future similar lockdowns on the adolescents' well-being..

## Highlights

• One in five adolescents reported economic disruptions during spring 2020 lockdown.
• Girls and older adolescents reported the worst social and mental well-being.
• Perceived change in sleep and physical activity varied by gender and behavior.
• Adolescents reported consuming more fruits and vegetables and less energy drinks.
• Findings can be used for time trends that include the lockdown period.

## 1 Introduction

In March 2020, the World Health Organization announced the spread of COVID-19 was a global pandemic (WHO, 2020). With a high degree of uncertainty about containment of the virus, many countries imposed national measures that focused on restrictions to day-to-day life such as closure of schools at rather short notice (World Bank, 2020). On the 11th March, shortly after the first confirmed cases of COVID-19 in Czechia, all schools were closed as part of the national lockdown restrictions (Czech Republic Ministry of Health, 2020). In Czechia, the spring 2020 national lockdown included the closure of restaurants, non-essential shops, gyms, and swimming pools. Travel was limited to work purposes, essential shopping and for taking care of close relatives. A few exceptions were allowed such as going outside for exercise or spending time in nature (Czech Republic Ministry of Health, 2020). Despite this national policy, little is known about how this has affected the lives of adolescents in Czechia.

When the schools were closed during the spring 2020 lockdown, the opportunities for peer face-to-face interactions were limited, and all-day residence under the care of parents resulted in changes to adolescent health behaviours (Wang et al., 2020). For example, families had to plan their shopping more meticulously, not only due to the fact that children stayed home all day, but also due to concerns about food availability (Benker, 2021). Similarly, the normal physical activity routines were affected without physical education, organized sport or use of sport facilities. More time spent in front of screens have resulted from restrictions limiting mobility and the volume of indoor activities increased (Mitra et al., 2020).

Another key challenge for adolescents during lockdown was the ability to cope with changes to lifestyles as a consequence of stay-at-home policies in place. Technology became an indispensable solution for adolescents for formal and informal communications. As such, a significant increase in daily screen time viewing hours creating sedentariness and risks for poor health has been reported elsewhere (Király et al., 2020). Moreover, there were signs of an overall deterioration of adolescent mental health during the spring 2020 lockdown (Duan et al., 2020, Guessoum et al., 2020), such as depression and anxiety symptoms after being exposed to COVID-19, exacerbated by low social support (Qi et al., 2020) and loneliness (Ellis et al., 2020). These findings seem to have been worse among girls and older adolescents (Racine et al., 2020).

In recent years, research literature indicates notable gender differences in health behaviors during adolescence (Inchley et al., 2020). For example, girls are more likely to report better eating habits (i.e., eating more fruits and vegetables, but drinking less soft drinks), whereas boys report higher levels of physical activity (PA) (Biddle et al., 2011) and sedentary behaviors (Ng et al., 2018). Yet, when it comes to changes in behaviors during lockdown, PA among girls increased in Germany and Canada during the lockdown (Moore et al., 2020, Schmidt et al., 2020) whereas, researchers in Croatia reported significant decreases among boys (Sekulic et al., 2020).

Furthermore, the perceptions of the outcomes afforded by health behavior changes depend on both the behavioral frequency and direction of changes in behavior an individual is considering (Kiviniemi and Rothman, 2008). Bringing these aspects together, as most of these health behaviors are context dependent, it is important to establish whether these gender differences remain stable in circumstances such as those elicited by a forced national lockdown. Secondly, the investigation of perceived changes may serve well as a proxy indicator of actual changes when longitudinal or panel data are not available. There are few studies from Central Europe on how the COVID-19 pandemic has impacted adolescent well-being and health behaviors. Therefore, the aim of this study is twofold. Firstly, we aim to report on the experiences (i.e. household disruptions; social and mental well-being, and perceived change in health behaviors) of adolescents in Czechia by gender and age during the spring 2020 lockdown when schools were closed. Secondly, we examine the interaction between adolescent health behaviors, perceived changes in health behaviors and gender during the spring 2020 lockdown.

## 2 Methods

### 2.1 Sample and procedure

The present study is linked to the Health Behaviour in School-aged Children (HBSC): a WHO collaborative cross-national study and followed its methodology (Inchley et al., 2018). In May 2020, 234 schools (International standard classification of education levels 1 and 2) across all 14 Czech administrative regions were invited to participate in the online survey on the impact of the lockdown on adolescent health. Out of them, 144 agreed to take part in the survey (response rate at the school level 61.5%). No special education schools were invited. In each school, adolescents enrolled in 5th, 7th and 9th grades (corresponding to age categories of 11, 13, and 15 years, respectively) were invited to participate in the survey. Data were collected online in June 2020 via internet links sent to pupils by their school teachers (n = 3,384) with the exception of data from four schools which opted for a pen-paper questionnaire (n = 232). The class teachers of the given grades were asked to distribute the questionnaire link to all the pupils in their classes to fill it at home. The overall response rate at the individual level was 19% and varied between 1 and 72% across the participating schools. Previous studies on differences between pen-and-paper and online surveys show no statistically significant differences in responses (Šmigelskas et al., 2019), and therefore all survey data were pooled. Participation in the study was anonymous and voluntary. The participants could withdraw from the survey at any moment or skip questions that made them feel uncomfortable. Active consent was obtained at school and pupil level, whereas passive consent was used at parent level. The study design was approved by Palacky University ethics committee under reg. no. 65/2020.

### 2.2 Survey instruments

#### 2.2.1 Background variables

Adolescents were asked to report their gender, grade at school, and the size of the municipality they lived in. Further, there were two separate questions on the perceived changes in time spent on schoolwork during lockdown and leisure activities on a five-point scale from (1) ‘definitely more’ to (5) ‘definitely less’ and were grouped into more, same or less. In addition, adolescents were asked if they or someone in their family had tested positive for COVID-19.

#### 2.2.2 Experiences during lockdown

##### 2.2.2.1 Household disruptions due to COVID

An updated version of the Perceived Impact of the 2008/09 Economic Crisis Scale (Due et al., 2019) to the context of the spring 2020 lockdown was used including: *economic disruptions* (2 items: parents losing their jobs; having less money in the house), *psychosocial disruptions* (2 items: more family disputes; more parental stress), and *opportunities* (2 items: learn new things; more time to engage in joint activities with family that they all enjoyed). Response options were ‘yes’, ‘no’, and ‘don’t know’ (recoded to “no”).

##### 2.2.2.2 Social and mental well-being during lockdown

Adolescents reported how often, during the spring lockdown, they: i) felt lonely, ii) felt that they were part of a group of friends, and iii) had people they could talk to about important things. Response options ranged from (1) ‘never’ to (5) ‘very often’. Items were adapted from the R-UCLA scale (Hughes et al., 2004) for adolescent use. Life satisfaction was measured using a single visual analogue scale (Cantril ladder) (Cantril, 1966). The scale ranges from (0) ‘worst possible life’ to (10) ‘best possible life’. A cut-off for very high life satisfaction was set to nine in line with a previous HBSC study (Due et al., 2019).

### 2.3 Health behaviors and their perceived changes compared to pre-lockdown period

Adolescents reported, on a five-point scale with responses ranging from (1) ‘definitely more’ to (5) ‘definitely less’, whether they engaged in the following health behaviours more or less compared to pre-lockdown: i) sleep during weekdays and ii) weekends; iii) time spent watching movies, videos or series on TV or the Internet; iv) using social media; v) gaming on PC, tablets, consoles, etc.; vi) browsing the Internet or searching for information on the Internet; vii) overall moderate-to-vigorous physical activity (MVPA); consumption of viii) fruit; ix) vegetables; x) sweets; xi) soft drinks; and xii) energy drinks.

Reports of the adolescents’ health behaviors were asked through standardized measures from the HBSC Study (Inchley et al., 2018) on screen time, MVPA, food (i.e. sweets, fruit and vegetables) and drink consumption, as well as sleep through time to bed and wake up on weekdays and weekends (Tynjälä, 1999). Items in relation to food and drink consumption (Inchley et al., 2020, Holubcikova et al., 2017), as well as physical activity, sleep, and screen time were dichotomized based on recommended cut-offs (Physical Activity Guidelines Advisory Committee, 2018, Bucksch et al., 2016, Paruthi et al., 2016). All items have been used previously in the Czech language and have been tested for the psychometric properties in previous HBSC data collection rounds. The items were slightly reworded to reflect the period of the spring 2020 lockdown.

### 2.4 Analyses

Descriptive statistics were computed based on gender. To explore gender or grade differences, contingency tables were performed on household disruptions and social and mental well-being variables by gender and grade, including the total dependency with the interaction with gender and age. The differences in perceived changes in health behaviors during the spring 2020 lockdown were analyzed by log-linear analyses with the interaction of gender. A backward elimination approach was used in the loglinear model, computed by IBM SPSS 27.0, whereby all three-way interactions were included in a saturated model. If there were sufficient differences across the 2 × 2 × 3 tables, then a three-way interaction was reported. When there was no three-way interaction, three two-way interactions were included (gender × perceived change, gender × reported behavior, reported behavior × perceived change) to identify what differences there were between groups.

## 3 Results

### 3.1 Population

Responses from adolescents in Czechia (n = 3,440, 54% girls mean age = 13.5 years, SD = 1.6) were included in the final data set. For each of the 14 regions of Czechia, the representativeness of the sample ranged between 10 and 120 per 100,000 students and half (54%) from cities with under 10,000 inhabitants. At the time up to data collection (i.e., June 2020), <1% of adolescents (n = 21) had reported they had a positive test with COVID-19 and over double (n = 47) reported someone from their family was diagnosed with COVID-19 (Table 1). Perceived increases in schoolwork were reported by half the adolescents (52%) and two thirds (66%) perceived they spent more time in leisure time activities.

**Table 1:** Sample descriptive statistics table by gender.

	Total %	Boys %	Girls %
N	3440	1574	1866
Age category			
11 years old (M = 11.00; SD = 0.48 years)	31.4	33.7	29.4
13 years old (M = 12.97; SD = 0.51 years)	38.8	40.0	37.8
15 years old (M = 15.01; SD = 0.47 years)	29.8	26.3	32.7
Place or residence			
<2000	31.7	30.3	32.9
2000–9,999	22.0	20.8	23.0
10,000–49,999	29.6	30.5	28.8
50,000 or more	16.8	18.5	15.3
School Type			
Grammar	6.8	6.0	7.5
Primary	93.2	94.0	92.5
COVID-19 Diagnosed			
Adolescent	0.6	0.7	0.5
Someone in family	1.4	1.3	1.4
Perceived change in time spend on schoolwork			
Less	25.6	26.9	24.5
Same	22.0	21.8	22.1
More	52.4	51.2	53.4
Perceived change in time available for leisure activities			
Less	14.0	14.1	13.9
Same	20.2	19.4	20.9
More	65.8	66.4	65.2

### 3.2 Household disruptions and socialization

One in five adolescents (19.3%) indicated their families experienced economic disruptions during the lockdown (Table 2). Yet, significantly more girls than boys (37% v 26%, p < .001) reported psychosocial disruptions to their family life and this increased by age only for girls (p < .001). Despite these negative aspects, opportunities for positive interactions within the family or space to learn new things were also commonly reported (79%), although this declined with increasing age (p < .001).

**Table 2:** Contingency table for gender and age for remote schooling, household disruptions and socialization during lockdown (N = 3440).

	Total	Total	Boys				Girls				Total	
	N	%	11y %	13y %	15y %		11y %	13y%	15y %		Age difference	Gender difference
			531	628	414	p^a^	549	706	611	p^b^	p^c^	p^d^
Household disruptions												
Economic disruption	3439	19.3	21.7	16.7	15.7	0.031	19.7	21.0	20.0	0.883	0.352	.113
Psychosocial disruption	3437	31.7	24.5	25.3	28.3	0.400	28.8	36.3	44.4	<.001	<.001	<.001
Positive opportunities	3439	78.7	83.6	79.6	70.5	<.001	85.8	80.5	70.5	<.001	<.001	.885
Social and mental well-being												
Feeling lonely	3418	14.4	7.4	8.0	14.7	<.001	12.1	18.1	24.5	<.001	<.001	<.001
Feeling part of a group	3357	36	35.5	42.7	40.8	0.044	30	34.5	36.9	0.043	0.006	0.001
Someone to talk to	3418	59.1	61.3	60.7	52.7	0.013	62.9	59.8	55.6	0.039	<.001	0.735
High life satisfaction	3380	37.3	46.4	40.8	35.5	0.003	47.9	33.0	22.8	<.001	<.001	<.001

*Note*: Statistical tests were Chi-square tests. p^a^ = Difference in boys among age groups, p^b^ = Difference in girls among age groups, p^c^ = between ages (combined gender), p^d^ = between gender (combined ages) Perceived changes in health behaviors during lockdown: interactions with gender and health behavior

Almost twice as many girls than boys (18% v 10%; p < .001) had regular feelings of loneliness during lockdown. Greater proportion of younger adolescents reported high life satisfaction, compared to their older peers, with the difference being especially pronounced in girls (p < .001). Moreover, over a third (36%) of our sample regularly had feelings of being part of a group of friends during the lockdown, with the youngest age category experiencing this feeling least frequently.

**Fig. 1:**
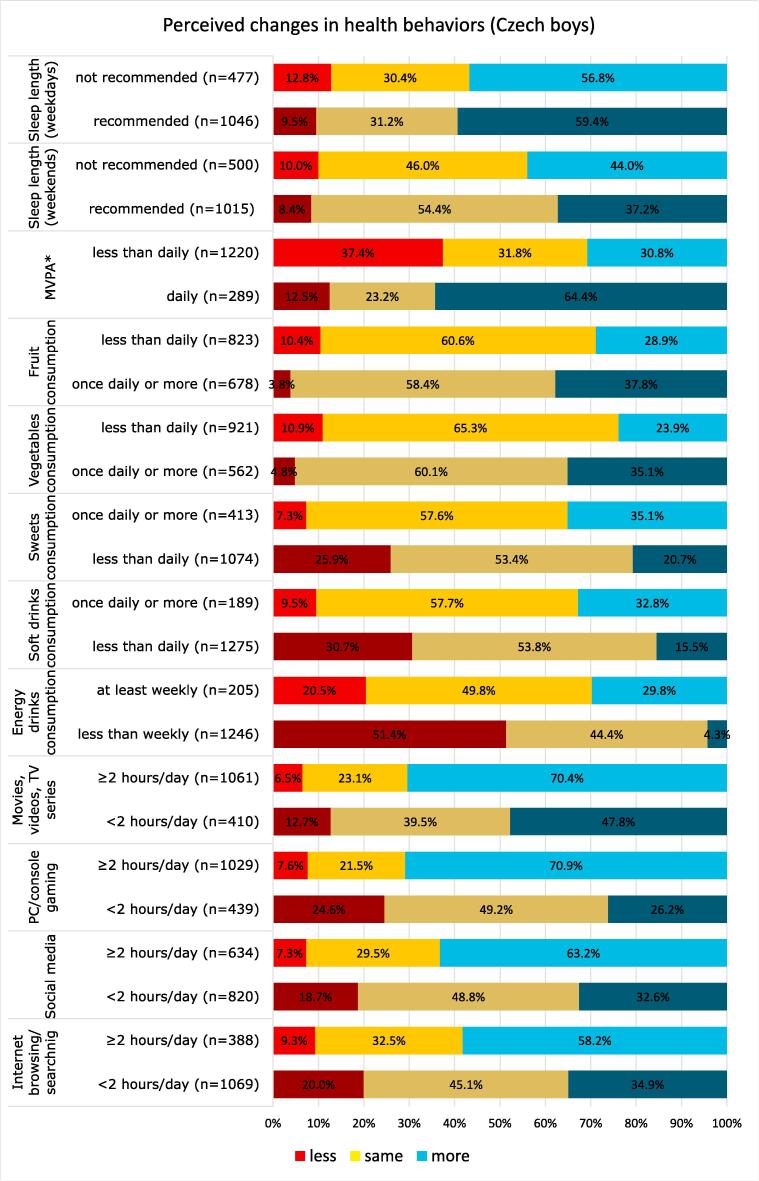
Boys’ perceived changes in health behavior by reported health behaviors. *MVPA – moderate-to-vigorous physical activity.

**Fig. 2:**
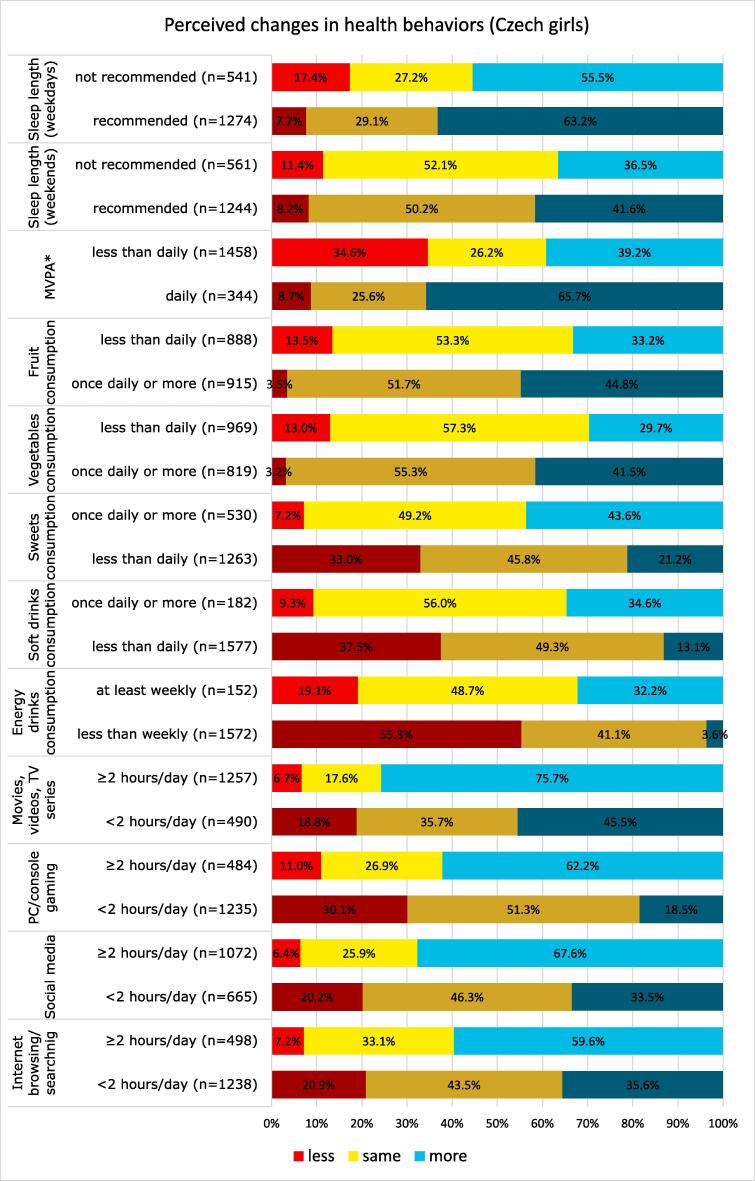
Girls’ perceived changes in health behavior by reported health behaviors *MVPA – moderate-to-vigorous physical activity.

Fig. 1, Fig. 2 outline the differences in perceived changes in health behaviors for boys and girls, respectively. The three-way interactions between gender, behavior and perceived behavior change were statistically significant only for sleep on weekdays nights (p = .039), weekend nights (p = .005) and physical activity (p = .04) (Table 3). Insofar, boys were 3.3 times more likely than girls to perceive they have slept less during the lockdown than those who did not sleep the recommended amounts and slept more than before the lockdown. Moreover, boys who reported daily MVPA were 2.8 times more likely to perceive they did more MVPA during the spring 2020 lockdown, and 1.7 times more than girls in the same group (daily MVPA and perceived to be more physically active), compared with the pre-lockdown period.

**Table 3:** Log-linear results on the interaction between gender, perceived behavior change, and reported behaviors.

	3 way		2 way					
	gender × behavior × perceived change		gender × behavior		gender × perceived change		behavior × perceived change	
	χ^2^	p	χ^2^	p	χ^2^	p	χ^2^	p
SleepSleep								
Weekdays	6.488	0.039	-		-		-	
Weekends	10.483	0.005	-		-		-	
Physical Activity								
Daily 60 min MVPA	6.427	0.04	-		-		-	
Food and Drink								
Daily Fruits	-		9.076	0.002	16.038	<.001	103.669	<.001
Daily Vegetables	-		17.624	<.001	15.885	<.001	102.38	<.001
Daily Sweet	-		-		20.05	<.001	267.981	<.001
Daily Soft Drinks	-		-		16.618	<.001	149.915	<.001
Weekly Energy Drinks	-		22.193	<.001	-		296.227	<.001
Screen time								
2h/day TV, Video series	-		-		12.388	0.002	203.786	<.001
2h/day Gaming	-		360.014	<.001	24.483	<.001	561.974	<.001
2h/day Social Media	-		84.446	<.001	-		348.262	<.001
2h/day Internet	-		-		-		168.117	<.001

*Note*: Gender = boys vs. girls, behavior = recommended vs. not recommended behavioral frequency, change = less, same, or more during lockdown. “-” association was not statistically significant.

Two-way interactions between gender and perceived behavioral change were observed in daily fruit, vegetable, sweet and soft drink and weekly energy drinks consumption, as well as screen-based activities. As such, more girls than boys reported eating more fruits and vegetables, and used social media, although more boys reported 2 h or more of gaming. Furthermore, based on the gender × perceived change interaction, more boys perceived their gaming time increased as well as screen time (e.g. TV, computer use), although more girls perceived an increase in daily fruit, vegetable, sweet and soft drink consumption. Reports of perceived changes of behavior based on the behavior itself was significant for all health behaviors.

## 4 Discussion

This is one of the first studies undertaken in Czechia that aimed to examine adolescents’ experiences and health behaviors during the COVID-19 related spring 2020 lockdown. Overall, less than a third of the participants reported disruptions to their family economic and psychosocial life. Girls and older adolescents reported the worst mental well-being, although, overall a series of positive perceived changes in adolescent health behaviors were reported (e.g., more physical activity and fruit and vegetable consumption or lower rate of energy drink consumption). Perceptions of changes in sleep and physical activity varied by gender, as well as level of these health behaviors during the lockdown. In addition, perceived changes in eating and drinking habits, as well as screen time activities varied considerably based the amounts of behavior.

Despite the sudden changes in their living circumstances, one in five adolescents reported economic disruptions, under a third reported psychological disruptions and three-quarters reported positive opportunities frequently. There were few reported cases or casualties compared to other countries (European Centre for Disease Prevention and Control, 2020), and perhaps the adolescents felt less endangered by the virus. This, altogether with a break from regular structures and routines provided the adolescents and their families with space to engage in activities they all enjoyed, but was also possibly related to low adherence to regulations (Residori et al., 2020). Two-thirds of the adolescents reported perceived increases in leisure time activities and half had perceptions they spent more time on schoolwork during lockdown. More strategies need to be developed to facilitate the continuation of enjoyable activities when schooling returns (Margaritis et al., 2020).

Around 30% adolescents reported eating sweets during the spring 2020 lockdown. Comparisons with previously collected representative data from 2018 (19–23%) (Inchley et al., 2020) would suggest there was an increase during spring 2020 lockdown. Similar findings were reported from findings in Italy, Spain, Chile, Columbia and Brazil (Ruiz-Roso et al., 2020). On the other hand, increases in eating daily fruits and vegetables were observed, as per the perceived changes described by respondents, but also by comparisons to data from the 2018 HBSC survey (Inchley et al., 2020). Similarly, over half the adolescents who drank less than one energy drink per week, suggested they consumed less than normal during the spring 2020 lockdown. These improvements in healthy eating habits were similar to earlier data reported (Whitehead et al., 2020) from Western Europe, Commonwealth countries or USA and could be attributed to the consumption of all meals at home, where choice of food and drinks may have been limited during lockdown since parents took responsibility of the dieting habits of all under the same roof (Benker, 2021). Energy drink consumption is also considered a socializing activity, whereby many of the adolescents drink them together with alcohol or during sport practices (Zucconi et al., 2013) and with the social restrictions in place, there were reduced opportunities to consume energy drinks. The results in this study can be useful when exploring time trends in adolescent health behaviors as the instruments used were the same as in previous, as well as planned data collection waves in the HBSC study. Future research could examine the changes in lockdowns and their impact on adolescent health and well-being (Mynaříková and Novotný, 2020).

As it might be expected, we found circumstances that were associated among adolescents with low mental well-being. Perceptions of poorer mental health of adolescents were more noticeable among girls than boys, as reported in other studies across the world (Guessoum et al., 2020, Qi et al., 2020). Moreover, being away from the school routine was linked with poor mental health (Racine et al., 2020, Lee, 2020) and uncertainties about COVID-19 among the Czech population generated much pessimism (Trnka and Lorencova, 2020), of which, could have been transferred to some adolescents too. We observed feelings of loneliness and not feeling part of a group increased with age, supporting existing knowledge about the increasing role of peer relationships increases on individual’s health (Patton et al., 2016). Given that the physical contact was removed during the spring lockdown (Residori et al., 2020, Oosterhoff et al., 2020), further studies are needed to find out how adolescents continue to interact with each other during subsequent lockdowns. Currently, there does not seem to be adequate knowledge or tools on how to promote adolescent mental health, despite it being a major public health issue (Lee, 2020). With the advancement of technology and need to adapt services, the adoption of tele-health services may be a useful future strategy given the restrictions to meet people were in force during the lockdown (Evans et al., 2020).

Our findings outline a gendered pattern in the associations between levels of health behaviors and their perceived change due to the lockdown. This was particularly the case for the changes in sleep and physical activity, and varied depending on whether the adolescent met the recommended amount of sleep or physical activity. For example, the girls who slept the recommended time on weekdays were more likely to report having slept for more compared with the pre-lockdown period than girls who did not sleep the recommended amount on weekdays or boys. Other researchers reported higher proportions of adolescents going to bed at later times during lockdown (Bruni et al., 2021) yet also waking up at later times (Becker et al., 2021). Similarly, boys who did not participate in 60 min of daily MVPA during lockdown were more likely to report they did less MVPA than boys who did MVPA daily or girls. The pattern of doing more PA and meeting a cut-off of daily 60 min of MVPA is reported widely (Moore et al., 2020, Ng et al., 2020, Chambonniere et al., 2021, Tulchin-Francis et al., 2021), and despite organized sport venues being restricted, it is likely that creative ways (at various intra-, interpersonal, environmental and policy levels) to sustain PA or even encourage higher PA levels have taken place in Czechia during the spring 2020 lockdown. The types of activities taken up during lockdown need investigating with opportunities to sustain them to reduce physical inactivity. Such findings require further investigations as regular physical activity is highly correlated with participation in sports during leisure time, and during lockdown, these opportunities were eliminated (Ng et al., 2020). The perceived increase in screen-time activities is likely due to remote schooling, online socializing and limited non-screen-based activities (Margaritis et al., 2020, Nagata et al., 2020). Based on these results, public health messages are needed to promote more physical activity and reinforce the potential harm of excessive screen-time activities, particularly if it replaces physical activities (Sallis et al., 2020) or sleep (Gába et al., 2020). In addition, the practical recommendations for parents to guide or regulate the amount of online behavior during the spring lockdown (Király et al., 2020) requires further monitoring to see what effects result from the restrictions following the pandemic.

There are some limitations to consider when interpreting the results from this study. The results are based on self-reported data in a cross-sectional study that relied upon recall over a very specific period of the spring 2020 lockdown. As with other published studies, the health behaviors instruments have been checked for suitability for use among young adolescents and are used frequently in national data collection (Inchley et al., 2018). There was not enough time to validate the new items in relation to disruptions due to COVID-19. Also, the sample was approached through schools that were willing to take part in the study at a time when schools were closed or reopening partly. Lastly, this is a snapshot of the particularities associated with the first wave of COVID-19 lockdown in Czechia. Given the escalation of the COVID-19 in late 2020 and early 2021, especially the increase in the number of cases, deaths and nationwide extended lockdown, these results are likely not representative for the latter situation and more research is needed to understand how all these later developments impacted adolescent health and well-being. Despite these study limitations, to our knowledge, this is the largest COVID-19 sample collected among young adolescents in Czechia. The well-established HBSC methodology, including the core questionnaire items, initial sampling procedures and key age categories were replicated as much as possible given the COVID-19 situation, that can be considered when analyzing and reporting time trends in adolescent health behaviors.

## 5 Conclusion

As part of the restrictions from the COVID-19 pandemic school were closed. GGGirls and older adolescents reported the worst outcomes in psychosocial disruptions and mental well-being. There were large discrepancies among Czech adolescents’ perceived changes in health behaviors during lockdown and with conforming to sleep, sedentary behaviors, physical activity, and energy drink recommendations. The estimates from this study may be useful to explain future time trend analyses and cross-national studies. Continued health promotion activities are needed to encourage healthy behaviors among adolescents, particularly during state of emergency circumstances.

## Declaration of Competing Interest

The authors declare that they have no known competing financial interests or personal relationships that could have appeared to influence the work reported in this paper.
